# Non-Sagittal Knee Joint Kinematics and Kinetics during Gait on Level and Sloped Grounds with Unicompartmental and Total Knee Arthroplasty Patients

**DOI:** 10.1371/journal.pone.0168566

**Published:** 2016-12-21

**Authors:** Igor Komnik, Markus Peters, Johannes Funken, Sina David, Stefan Weiss, Wolfgang Potthast

**Affiliations:** 1 Institute for Biomechanics and Orthopaedics, German Sport University Cologne, Cologne, Nordrhein-Westfalen, Germany; 2 ARCUS Clinics Pforzheim, Pforzheim, Baden-Württemberg, Germany; University of Memphis, UNITED STATES

## Abstract

After knee arthroplasty (KA) surgery, patients experience abnormal kinematics and kinetics during numerous activities of daily living. Biomechanical investigations have focused primarily on level walking, whereas walking on sloped surfaces, which is stated to affect knee kinematics and kinetics considerably, has been neglected to this day. This study aimed to analyze over-ground walking on level and sloped surfaces with a special focus on transverse and frontal plane knee kinematics and kinetics in patients with KA. A three-dimensional (3D) motion analysis was performed by means of optoelectronic stereophogrammetry 1.8 ± 0.4 years following total knee arthroplasty (TKA) and unicompartmental arthroplasty surgery (UKA). AnyBody^™^ Modeling System was used to conduct inverse dynamics. The TKA group negotiated the decline walking task with reduced peak knee internal rotation angles compared with a healthy control group (CG). First-peak knee adduction moments were diminished by 27% (TKA group) and 22% (UKA group) compared with the CG during decline walking. No significant differences were detected between the TKA and UKA groups, regardless of the locomotion task. Decline walking exposed apparently more abnormal knee frontal and transverse plane adjustments in KA patients than level walking compared with the CG. Hence, walking on sloped surfaces should be included in further motion analysis studies investigating KA patients in order to detect potential deficits that might be not obvious during level walking.

## Introduction

In recent years, studies in the field of KA research have shown abnormal gait characteristics during various activities of daily living (ADL)[1–3]. However, previous authors primarily investigated level walking in patients after TKA surgery, revealing reduced knee flexion excursion and knee flexion moment during the loading response phase compared with healthy controls [4,5]. Physically more demanding ADL, such as stair climbing tasks, showed inconsistent results in terms of kinematics and kinetics due to methodological flaws [6]. In general, biomechanical analysis of physically more demanding ADL, other than level walking, has been addressed less often in the previous literature. In this regard, there are no motion analysis studies to this day investigating KA patients negotiating sloped surfaces [7]. These kinds of daily barriers are common in urban areas and pose a challenge to the musculoskeletal system. Healthy subjects show more ankle, knee and hip joint flexion during incline walking compared with level walking, whereby greater demands occur in the hip [8–10]. On the other hand, movement adjustments occur particularly at the knee joint during decline walking, revealing increased eccentric knee extensor activity accompanied by enhanced mechanical power requirement in the stance phase [10,11]. Furthermore, declined surfaces increase the risk of slips and falls [12]. Hence, ramp negotiation could expose potential gait deficits which are more apparent and meaningful than during level walking in patients after KA surgery. This could be of great importance for the evaluation of KA patients’ rehabilitative progress.

However, besides the locomotion task, different types of prosthesis designs are considered to have a significant effect on patients’ knee kinematics and kinetics. Andriacchi et al. [1] emphasized the importance of cruciate ligament preservation in TKA which plays a major role concerning proprioceptive joint control. UKA assumes the preservation of both cruciate ligaments whereby, in contrast to TKA, only medial or lateral femur and tibia compartments are replaced. Several *in vitro* and *in vivo* studies based on fluoroscopic investigations reported that UKA succeeds at restoring normal joint kinematics during kneeling tasks [13–15]. Preservation of the anterior cruciate ligament (ACL), however, seems to be a major reason for the normal movement pattern of the replaced condyles. Further potential reasons for normal knee kinematics and kinetics in UKA patients, such as the preservation of the lateral knee compartment or different bearing shapes compared with TKA, should be considered. Addressing the question of whether UKA is able to restore normal knee mechanics compared with TKA, UKA patients in a study by Jung et al. [16] performed stair climbing with similar knee internal rotation in their operated knee to the non-operated knee (non-OP knee) in contrast to TKA patients. A recent study [17] used a machine learning approach to classify and identify UKA and TKA knees on the basis of recognized patterns. When applied to healthy controls their decision tree algorithm classified 92% of the healthy controls as being UKA subjects, revealing a more physiological gait than TKA subjects.

There has been a rising popularity of UKA in recent years and the aspect of a more natural gait pattern has contributed to this popularity. Nevertheless, UKA still represents a small proportion (8%) of all performed KAs [18]. Interestingly, studies have reported that 36%–48% of all candidates fulfill the criteria for UKA [19,20]. The potential functional benefit of UKA has not been studied sufficiently by means of comprehensive motion analysis, particularly under the consideration of physically more demanding ADL and inclusion of TKA and UKA groups as well as healthy controls [7]. A better understanding is necessary for better patient-specific preoperative planning in daily clinical practice and could provide decision support regarding the appropriate treatment.

Additionally, in the field of KA previous biomechanical studies have primarily focused on sagittal plane parameters. Frontal plane and particularly transverse plane kinematics and kinetics have by and large been neglected, although the clinical relevance of forces and moments acting in these planes was highlighted, especially in the field of osteoarthritis [21,22]. In this regard, studies clarified that knee adduction and internal rotation moments are significant discriminatory factors of osteoarthritic gait pattern. Furthermore, it has been shown that patients with ACL-deficient knees reveal abnormal tibial rotation in the transverse plane during gait and highly functionally demanding tasks, as the ACL restrains the tibia’s anterior-posterior translation, but also its internal rotation [23–25]. Moreover, aseptic loosening of prosthesis components remains, among others, the leading reason for failure [26,27]. In this regard, mechanical aspects, such as torsional micromotion contributing to component loosening, have not yet been adequately explained [28]. Investigation of transverse plane mechanics might provide better insight into factors leading to KA failure. Therefore, non-sagittal plane knee kinematics and kinetics are of particular interest concerning the influence of different locomotion tasks and prosthetic design.

The purpose of this retrospective case control study was to analyze knee kinematics and kinetics of patients with TKA and UKA with a special focus on the frontal and transverse plane during level, incline and decline walking compared with a healthy CG. The first hypothesis was that the UKA group would reveal more similar knee mechanics to the CG than to the TKA group. The second hypothesis was that decline walking in particular exposes greater abnormal kinematics and kinetics in the knee joint compared with level and incline walking in either KA group.

## Methods

### Ethics statement

Ethical approval for the current study was granted by The Ethics Committee of the German Sport University (ethical proposal no. 025/2014). All subjects signed a written informed consent form according to The Declaration of Helsinki.

### Participants

Thirty-seven subjects participated in the current study. All patients who underwent primary unilateral UKA and TKA for knee degenerative osteoarthritis were recruited from ARCUS Clinics Pforzheim (Germany). Initially, 210 TKA and 83 UKA patients were assessed for eligibility. Forty-nine subjects consented to participate in the current study. After a telephone interview 25 subjects had to be excluded. Ultimately, eleven patients formed the TKA-group and 13 subjects represented the UKA group. A healthy age matched CG consisted of 13 subjects who reported no knee pain and functional impairments for a period of one year prior to testing. Exclusion criteria were (1) further joint arthroplasties, (2) musculoskeletal impairments that affected ADL, (3) pain or functional impairment in the non-OP knee, (4) body mass index (BMI) greater than 31 kg/m^2^, (5) cardiovascular disease, (6) neurological disorders, (7) diabetes mellitus, (8) rheumatic diseases, limb-valgus deformity greater than 7° and limb-varus deformity greater than 4° and (9) knee flexion contracture greater than 5°. All UKA patients received a medial cemented endoprosthesis (Unicompartmental High Flex Knee System, Zimmer, Warsaw, USA). Seven TKA patients received a cemented posterior stabilized endoprosthesis (SIGMA, DePuySynthes, Warsaw, USA) and four patients received a cemented posterior cruciate ligament preserved endoprosthesis (Genesis II, Smith and Nephew, Memphis, USA). A minimally invasive approach was used in all UKA patients and a standard medial parapatellar approach in all TKA patients. Gait analysis was performed on average 1.8 ± 0.4 years post-surgery.

### Data acquisition

Gait analysis was performed by means of a 3D, 10-camera (100 Hz) motion analysis system (VICON MX40, Vicon Motion Systems Ltd, UK). Simultaneously, ground reaction forces were collected at 1000 Hz using three Kistler force plates (KistlerInstrumente AG, CH). Two force plates were embedded in the floor and one under a three-step ramp (gradient: 21%). In order to create a lower-limb model consisting of nine rigid segments, 28 spherical, retroreflective markers were attached bilaterally to the anterior and posterior superior iliac spines, greater trochanters, lateral and medial condyles of the femurs, tibias, lateral and medial malleoli, heads of the first and fifth metatarsals, second proximal phalanges, lateral, medial and backside of the calcanei. Data collection started with a standing reference trial. Subsequently, the CG, which was tested before both KA groups, practiced level walking on a 15-m walkway. The captured valid trials were within 5% deviation of the practiced speed. After several practice trials TKA and UKA subjects performed the level walking task at the CG’s comfortable average gait velocity of 1.4 m/s ± 5%. Following level walking, all subjects performed incline or decline walking tasks respectively. The order was randomly chosen by the examiners. In order to avoid impairments of subjects' habitual gait pattern, all subjects were asked to negotiate incline and decline walking at their comfortable speed. Gait velocity was measured by means of a time-gate system (WEKO, Weitmann & Konrad GmbH & Co KG., DE) and subsequently calculated by means of the center of mass (CoM) course. A trial was valid if a clear contact with only one foot on the appropriate force plate was detected. The subjects were asked to tell the examiners if they needed a rest or felt any discomfort or pain during the measurements. All participants accomplished the locomotion tasks free of pain.

### Data analysis

Kinematic and kinetic raw data were filtered by means of a recursive Butterworth low-pass filter at a 6-Hz cutoff frequency. The Anybody^™^ Modeling System (AnyBody Technology, Aalborg, DK) was used to perform lower-limb inverse dynamics according to the anatomical landmark scaled model of Lund et al. [29]. Standing reference trials were recorded for each subject to create a stick-figure model that was used to scale a cadaver dataset into subject-specific joint parameters. The defined knee joint coordinate system was based on Pennock and Clark [30]. The computed angles from standing reference trials were subtracted from the appropriate dynamic captured trials. The modeled head and trunk were driven by the pelvis markers. In order to determine inertial properties more accurately, subjects’ whole-body anthropometrics were measured to adjust the inverse dynamic model. The mass of a segment was assumed to be the product of the volume of a frustum and the segment’s density [31]. The knee joint was modeled as a spherical joint including three degrees of freedom, which were constrained using Anybody^™^’s Force-Dependent Kinematics method. A simple muscle model was used with third degree polynomial muscle recruitment. Kinematic and kinetic data were time-normalized to the stand phase, whereby the average of five valid trials was used for the analysis. KA patients’ operated limbs were compared with the right limbs of the CG. Further data processing was conducted with custom-built Matlab (2013b) routines (The MathWorks, Natick, USA). Knee biomechanics were assessed through the use of 3D angles and moments including peak varus angles, range of motion (RoM), peak adduction moments and knee adduction moment impulses within the first 50% of the stance phase. Peak angles, RoM and moments were extracted from the entire stance phase in the transverse plane. Joint moments are presented as external moments normalized to each subject’s mass and height. In order to detect increased lateral trunk sway indicating a potential compensatory mechanism, the distance between the ground projected CoM and the force application point (FAP) was calculated in the medial-lateral direction at the moment of the first peak knee adduction moment (FAP-CoM_add_). The distance values were normalized to each subject’s height. Knee joint stiffness (K_stiff_) was calculated in the transverse plane as the change in rotational knee joint moment (M), divided by the change in transverse plane knee joint angle (θ) between the initial ground contact and the instant of time when the knee joint was maximally internally rotated.

$${\text{K}}_{\text{stiff}}=\frac{\Delta \text{M}}{\Delta \theta }$$

The step length and width were normalized to each subject’s height. Sagittal plane kinematics and kinetics were measured but are not presented nor discussed in the current article. The authors added for the interested reader sagittal plane results as supporting information in the form of the S1 and S2 Tables.

### Statistical analysis

A test for normal distribution was performed using the Shapiro-Wilk Test. If the variables were normally distributed, univariate ANOVA was used to examine group differences. Individual between-group differences were clarified by means of a post hoc Tukey or Games-Howell test if the condition of homogeneity of variances was not fulfilled. In order to determine the interlimb asymmetry in the TKA and UKA group, the paired t-test was used. If variables were not normally distributed, the non-parametric Kruskal-Wallis Test was performed to detect between-group differences as well as the Wilcoxon-signed-rank test to determine interlimb asymmetries. The alpha level was set at 0.05 to detect a significant difference in all statistical tests. Effect size was calculated using eta-squared (η^2^) for ANOVA tests or Cohen’s d for interlimb comparisons. The point-biserial correlation (r_pb_) was used to determine the effect size for nonparametric tests [32]. An effect size value of 0.5 represents a large effect, 0.3 a medium effect and 0.1 a small effect [33]. Power analysis was conducted by means of the G*Power-software (G*Power Version 3.1.9.2, Kiel, Germany) in order to estimate the Type II error between the three investigated groups. All statistical tests were performed using IBM SPSS Statistics for Windows, Version 23.0 (IBM Corp., New York, USA).

## Results

### Subjects’ characteristics and time-distance parameters

Both KA groups weighed significantly more than the CG accompanied by significantly greater BMI values. Further characteristics of all subjects are listed in Table 1 as mean values ± standard deviation. No significant group differences were detected concerning time-distance parameters (contact time, step length, step width, and walking velocity).

**Table 1:** Subjects' characteristics and spatial-temporal parameters.

	Group	Mass [kg]	Height [m]	BMI [kg/m^2^]	Age	Male/female	Operated knee
	CG	68.7 ± 10.4	1.67 ±0.1	23.6 ± 2.7	57.6 ± 6	6/7	
	TKA	82.1 ± 9.7*^CG-TKA^	1.73 ± 0.1	27.5 ± 1.9*^CG-TKA^	60 ± 8.8	7/4	4 left/7 right
	UKA	80.4 ± 9.6*^CG-UKA^	1.7 ± 0.1	27.7 ± 2*^CG-UKA^	60.5 ± 7.8	7/6	5 left/8 right
**Task**		**Velocity [m/s]**	**Contact time [s]**	**Step length[cm]**	**Step width[cm]**		
Level walking	CG	1.4 ± 0.18	0.66 ± 0.06	37.3 ± 7.2	4.6 ± 1.5		
	TKA	1.4 ± 0.03	0.68 ± 0.03	39.1 ± 2.3	4.6 ± 1.2		
	UKA	1.4 ± 0.03	0.68 ± 0.03	38.9 ± 1.5	4.7 ± 1.7		
Decline walking	CG	1.2 ± 0.13	0.62 ± 0.06	29.5 ± 3.1	8.0 ± 2.9		
	TKA	1.2 ± 0.15	0.65 ± 0.06	29.3 ± 4.1	7.1 ± 2.2		
	UKA	1.1 ± 0.14	0.63 ± 0.04	27.8 ± 3.4	6.9 ± 0.7		
Incline walking	CG	1.2 ± 0.11	0.71 ± 0.06	40.9 ± 7.5	7.3 ± 2.8		
	TKA	1.2 ± 0.14	0.77 ± 0.06	40.0 ± 5.6	5.8 ± 2.6		
	UKA	1.2 ± 0.14	0.76 ± 0.06	40.0 ± 4.7	5.9 ± 1.7		

*Indicates a significant difference between corresponding groups.

### Transverse plane

No statistically significant differences were detected between the TKA and UKA groups, regardless of the locomotion tasks and parameters investigated (Tables 2 and 3).

**Table 2:** Between-group differences in peak knee joint angles, moments, adduction moment impulses, transverse joint stiffness and FAP-CoM_add_ during level walking.

	Locomotion task					
	Level walking					
Parameter	Group					
Angle [°]	CG	TKA	UKA	p-value	Effect size	Power
Varus	2.4 ± 1.6	2.4 ± 0.8	2.4 ± 1.4	0.897	0.01	0.05
RoM	2.3 ± 1.5	1.9 ± 0.6	2.1 ± 1.2	0.976	0	0.05
Int. rotation	3.0 ± 4.4	0.49 ± 2.8	4.5 ± 5.4	0.103	0.13	0.1
RoM	7.8 ± 2.7	11.3 ± 3.2	11.0 ± 5.4	0.062	0.16	0.12
Moment[Nm/(kg·m)]						
Adduction	0.28 ± 0.05	0.27 ± 0.05	0.32 ± 0.08	0.109	0.12	0.09
Internal rotation	0.07 ± 0.03	0.07 ± 0.02	0.09 ± 0.04	0.378	0.08	0.07
Adduction mom. impulse [Nms/(kg·m)]	0.05 ± 0.02	0.04 ± 0.01	0.05 ± 0.02	0.101	0.13	0.10
Joint stiffness [Nm/°]	0.008 ± 0.002	0.006 ± 0.001	0.007 ± 0.002	0.068	0.15	0.11
FAP-CoM_add_ [cm]	32.1 ± 8.0	33.9 ± 8.4	35.6 ± 5.6	0.503	0.04	0.05

**Table 3:** Between-group differences in peak knee joint angles, moments, adduction moment impulses, transverse joint stiffness and FAP-CoM_add_ during walking on a sloped surface.

	Locomotion task											
	Decline walking						Incline walking					
Parameter	Group						Group					
Angle [°]	CG	TKA	UKA	p-value	Effect size	Power	CG	TKA	UKA	p-value	Effect size	Power
Varus	2.7 ± 3.1	2.0 ± 1.4	1.8 ± 1.3	0.549	0.04	0.05	6.5 ± 4.0	6.2 ± 2.7	5.8 ± 2.5	0.838	0.01	0.05
RoM	3.5 ± 2.2	2.3 ± 1.1	2.3 ± 1.2	0.112	0.12	0.09	5.5 ± 2.9	4.8 ± 2.3	4.0 ± 2.1	0.3	0.07	0.06
Int. rotation	1.8 ± 6.6	-5.0 ± 3.2	-2.6 ± 2.9	0.012*^CG_TKA^0.109 ^CG-UKA^0.168 ^TKA-UKA^	0.28	0.29	2.2 ± 9.1	-1.2 ± 3.4	2.4 ± 5.0	0.399	0.05	0.06
RoM	7.1 ± 3.3	6.0 ± 2.0	6.0 ± 2.9	0.529	0.04	0.05	6.4 ± 4.4	8.9 ± 2.8	9.1 ± 3.5	0.027*^CG-TKA^0.030*^CG-UKA^0.839 ^TKA-UKA^	0.19	0.15
Moment [Nm/(kg·m)]												
Adduction	0.37 ± 0.06	0.27 ± 0.06	0.29 ± 0.07	0.001*^CG-TKA^0.007*^CG-UKA^0.753 ^TKA-UKA^	0.36	0.45	0.25 ± 0.07	0.25 ± 0.13	0.30 ± 0.10	0.45	0.05	0.06
Int. rotation	0.07 ± 0.05	0.06 ± 0.02	0.07 ± 0.03	0.93	0	0.05	0.03 ± 0.02	0.05 ± 0.04	0.07 ± 0.05	0.134	0.11	0.08
Adduction mom. impulse [Nms/(kg·m)]	0.06 ± 0.01	0.05 ± 0.01	0.05 ± 0.02	0.093 ^CG-TKA^0.072 ^CG-UKA^0.997 ^TKA-UKA^	0.14	0.10	0.04 ± 0.02	0.05 ± 0.03	0.06 ± 0.02	0.278	0.01	0.05
Joint stiffness [Nm/°]	0.01 ± 0.01	0.02 ± 0.01	0.06 ± 0.17	0.46	0.05	0.06	0.009 ± 0.006	0.005 ± 0.002	0.006 ± 0.002	0.2	0.09	0.07
FAP-CoM_add_ [cm]	60.0 ± 16.1	60.5 ± 13.6	58.9 ± 10.9	0.247	0	0.05	64.72 ± 13.2	50.2 ± 13.9	49.9 ± 14.0	0.019*^CG-TKA^0.038*^CG-UKA^0.931 ^TKA-UKA^	0.19	0.15

*Indicates a significant difference between corresponding groups.

**Fig 1:**
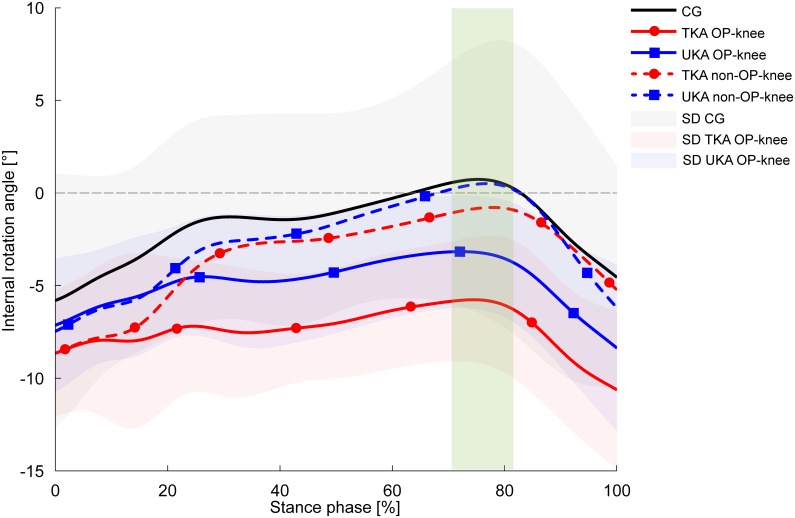
Knee internal rotation angles during decline walking. Values are presented as mean curves (solid lines) ± standard deviations (SD, shaded areas). Positive values indicate internal rotation. Dotted lines represent the non-OP knee of the TKA group (red) and UKA group (blue). The green rectangle indicates significantly different peak values between the TKA-OP knee and the CG (*p* = 0.012), the TKA-OP knee and TKA non-OP knee (*p* = 0.002), the UKA-OP knee and UKA non-OP knee (*p* = 0.007).

**Fig 2:**
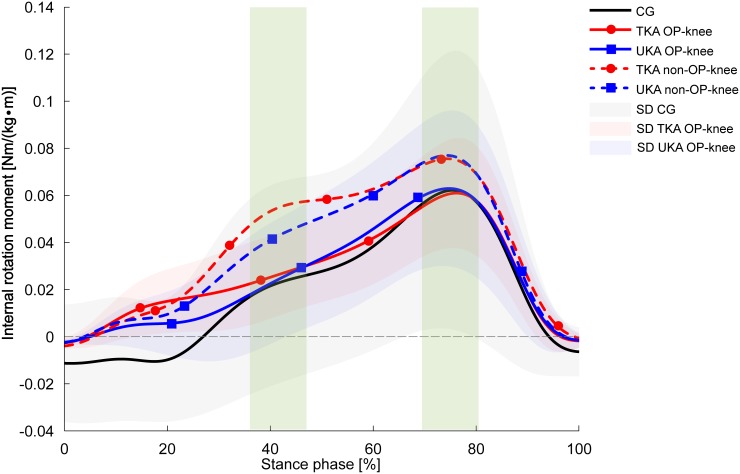
Knee internal rotation moments during decline walking. Values are presented as mean curves ± standard deviations (SD, shaded areas). Positive values indicate internal rotation moments. Dotted lines represent the non-OP knee of the TKA group (red) and UKA group (blue). The green rectangles indicate significantly different peak values between the TKA-OP knee and TKA non-OP knee, the UKA-OP knee and UKA non-OP knee for the first 50% of the stance phase (TKA: *p* = 0.002, UKA: *p* = 0.003) as well as 50%–100% of the stance phase (TKA: *p* = 0.041, UKA: *p* = 0.015).

The TKA and UKA groups showed considerably lower peak internal rotation values for the operated knee compared with the CG during decline walking [6.8° (TKA, *p* = 0.012) and 4.4° (UKA)], whereby the difference was statistically significant only between the TKA group and the CG (Fig 1). The effect size of 0.28 indicates a moderate effect. Concerning the interlimb differences, the operated (OP) knees in the KA groups revealed significantly diminished peak internal rotation values than the non-OP knees which presented similar values to those in the CG (1.8 ± 6.6°). The peak knee internal rotation moments were nearly equal in all groups (Fig 2). In contrast, both KA groups revealed significant 25% (TKA, *p* = 0.041) and 12.5% (UKA, *p* = 0.015) increases in internal rotation moments in their non-OP knees in comparison with the OP knees.

Furthermore, appreciable two-fold (TKA group) and six-fold (UKA group) higher joint stiffness values were observed in both KA groups for the OP knee compared with the non-OP knee during decline walking (Table 4). However, the difference was statistically significant only for the TKA group, whereas r_pb_ values (TKA = 1.12, UKA = 0.54) indicate a meaningful difference between both KA groups and the CG. No significant difference was detected between the non-OP knee and the CG regarding joint stiffness.

**Table 4:** Interlimb differences in peak angles, moments, adduction moment impulses, transverse joint stiffness, FAP-CoM_add_ within the TKA and UKA-group.

		Group							
Locomotion Task	Parameter	TKA				UKA			
	Angle [°]	Op	Non op	p-value	Effect size	Op	Non op	p-value	Effect size
Level walking	Varus	2.4 ± 0.8	3.6 ± 1.7	0.036	0.9	2.4 ± 1.4	4.0 ± 1.1	0.019	0.68
	ROM	1.9 ± 0.6	3.2 ± 1.2	0.01	1.4	2.1 ± 1.2	3.5 ± 1.1	0.03	1.22
	Int. rotation	0.49 ± 2.8	2.8 ± 3.3	0.003	0.76	4.5 ± 5.4	5.9 ± 4.4	0.375	0.28
	ROM	11.3 ± 3.2	7.7 ± 3.0	0.018	1.16	11.0 ± 5.4	11.2 ± 5.5	0.807	0.04
Delcine walking	Varus	2.0 ± 1.4	4.1 ± 2.4	0.016	1.07	1.8 ± 1.3	3.7 ± 1.9	0.008	1.17
	ROM	2.3 ± 1.1	3.4 ± 1.6	0.061	0.8	2.3 ± 1.2	3.4 ± 1.5	0.035	0.78
	Int. rotation	-5.0 ± 3.2	0.1 ± 5.6	0.002	1.12	-2.6 ± 2.9	1.1 ± 4.1	0.007	1.04
	ROM	6.0 ± 2.0	6.4 ± 2.9	0.772	0.16	6.0 ± 2.9	7.3 ± 3.3	0.16	0.42
Incline walking	Varus	6.2 ± 2.7	8.4 ± 3.1	0.021	0.76	5.5 ± 2.7	7.8 ± 3.7	0.572	0.71
	ROM	4.8 ± 2.3	3.8 ± 2.5	0.446	0.42	4.2 ± 2.2	5.0 ± 3.0	0.807	0.3
	Int. rotation	-1.2 ± 3.4	0.4 ± 3.3	0.058	0.48	3.2 ± 4.3	1.5 ± 4.7	0.187	0.38
	ROM	8.9 ± 2.8	6.6 ± 1.8	0.023	0.98	9.2 ± 3.7	8.7 ± 2.8	339	0.15
	Moment [Nm/(kg·m)]								
Level walking	Adduction	0.27 ± 0.05	0.31 ± 0.12	0.363	0.43	0.32 ± 0.08	0.36 ± 0.05	0.171	0.6
	Int. rotation	0.07 ± 0.02	0.08 ± 0.04	0.41		0.09 ± 0.04	0.10 ± 0.02	0.48	0.32
Decline walking	Adduction	0.27 ± 0.06	0.33 ± 0.15	0.299	0.53	0.29 ± 0.07	0.33 ± 0.07	0.086	0.57
	Int. rotation	0.06 ± 0.02	0.08 ± 0.04	0.041	0.62	0.07 ± 0.03	0.08 ± 0.03	0.015	0.33
Incline walking	Adduction	0.25 ± 0.13	0.32 ± 0.1	0.164	0.6	0.28 ± 0.1	0.36 ± 0.1	0.824	0.8
	Int. rotation	0.05 ± 0.04	0.07 ± 0.03	0.182	0.57	0.07 ± 0.05	0.08 ± 0.04	0.439	0.22
Level walking	Adduction mom. impulse[Nms/(kg·m)]	0.05 ± 0.01	0.05 ± 0.03	0.51	0	0.06 ± 0.02	0.06 ± 0.01	0.556	0
Decline walking	Adduction mom. impulse [Nms/(kg·m)]	0.05 ± 0.01	0.06 ± 0.03	0.376	0.45	0.05 ± 0.02	0.06 ± 0.01	0.113	0.63
Incline walking	Adduction mom. impulse [Nms/(kg·m)]	0.05 ± 0.03	0.07 ± 0.02	0.146	0.78	0.06 ± 0.02	0.08 ± 0.02	0.61	1
Level walking	Joint stiffness [Nm/°]	0.006 ± 0.001	0.005 ± 0.002	0.187	0.63	0.007 ± 0.002	0.006 ± 0.002	0.55	0.5
Decline walking	Joint stiffness [Nm/°]	0.020 ± 0.014	0.010 ±0.003	0.008	0.8	0.065 ± 0.173	0.011 ± 0.003	0.06	0.54
Incline walking	Joint stiffness [Nm/°]	0.005 ± 0.002	0.007 ± 0.003	0.041	0.62	0.006 ± 0.003	0.008 ± 0.004	0.36	0.57
Level walking	FAP-CoM_add_ [cm]	33.7 ± 8.4	34.4 ± 6.6	0.814	0.09	35.5 ± 5.5	33.9 ± 7.1	0.594	0.25
Decline walking	FAP-CoM_add_ [cm]	60.5 ± 13.6	56.9 ± 16.1	0.364	0.24	58.9 ± 10.9	54.1 ± 9.3	0.192	0.47
Incline walking	FAP-CoM_add_ [cm]	50.2 ± 13.9	48.4 ± 15.0	0.764	0.12	49.9 ± 14.0	46.4 ± 16.7	0.657	0.23

The TKA group tended to accomplish level walking with decreased peak internal knee rotation in comparison with the UKA group and CG (CG: 3.0° ± 4.4; TKA: 0.49° ± 2.8; UKA: 4.5° ± 5.4; *p* = 0.103). Nevertheless, the interlimb comparison revealed a slight but significant decrease of 2.3° (*p* = 0.003, d = 0.76) peak internal rotation of the OP knee in the TKA group.

As shown in Table 3, similar results to those obtained with level walking were observed during the incline walking task, except for the RoM which was significantly greater in both KA groups compared with the CG.

### Frontal plane

No statistically significant differences were detected between the TKA and UKA group considering the investigated locomotion tasks and parameters. The static knee varus angles extracted from the KA subjects’ standing reference trials showed neither statistically significant intergroup (TKA: −3.6° ± 1.7; UKA: −1.7° ± 3.4, *p* = 0.90, d = 0.7) nor interlimb differences (TKA op: −3.6° ± 1.7; TKAnon op: −2.7° ± 4.4; *p* = 0.37, d = 0.27; UKA op: −1.66° ± 3.4; UKAnon op: −2.1° ± 3.0; *p* = 0.73; d = 0.14).

**Fig 3:**
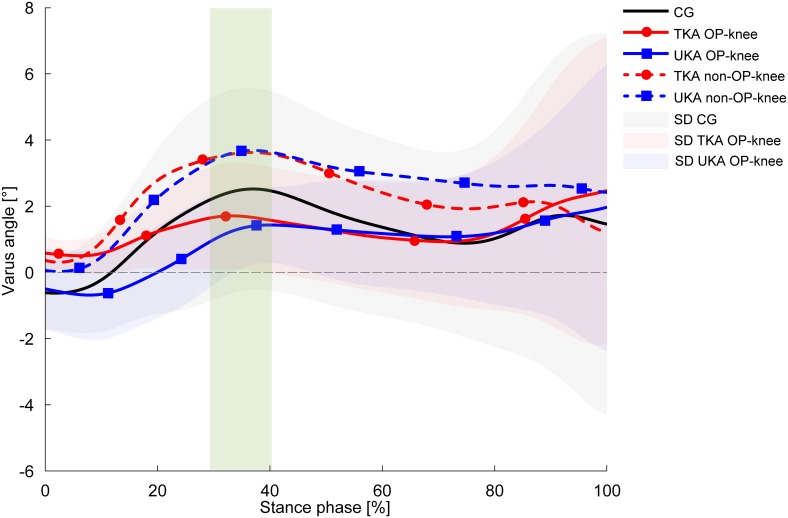
Knee varus angles during decline walking. Values are presented as mean curves ± standard deviations (SD, shaded areas). Positive values indicate varus alignment. Dotted lines represent the non-OP knee of the TKA group (red) and UKA group (blue). The green rectangle indicates significantly different peak values between the TKA-OP knee and TKA non-OP knee (*p* = 0.016), the UKA-OP knee and UKA non-OP knee (*p* = 0.008) for the first 50% of the stance phase.

**Fig 4:**
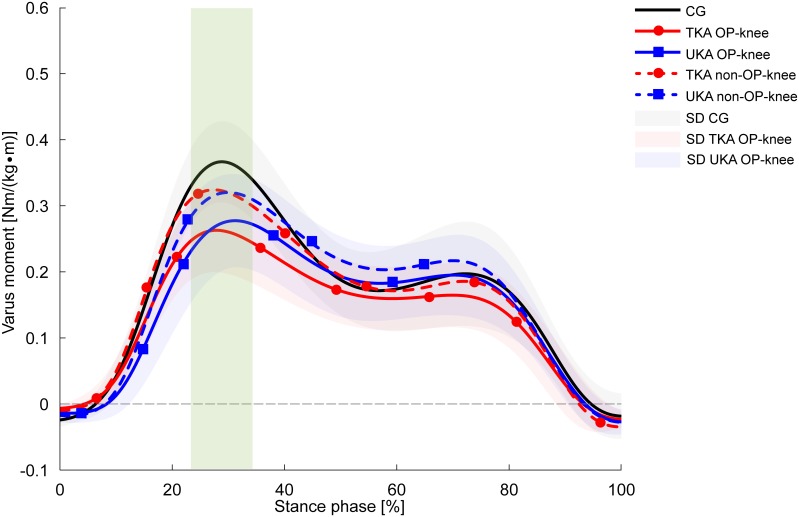
Knee adduction moments during level walking. Values are presented as mean curves (solid lines) ± standard deviations (SD, shaded areas). Positive values indicate adduction moments. Dotted lines represent the non-OP knee of the TKA group (red) and UKA group (blue). The green rectangle indicates significantly different peak values between the TKA-OP knee and CG (*p* = 0.001), the UKA-OP knee and CG (*p* = 0.007).

According to Table 4, both KA groups conducted their trials with greater peak knee varus angles in the non-OP knee compared with the OP knee, regardless of the locomotion task. However, the decline walking task revealed the most substantial discrepancies showing, e.g., greater RoM in the non-OP knee as well as 2.1° (TKA, *p* = 0.016, d = 1.07) and 1.9° (UKA, *p* = 0.008, d = 1.17) greater peak knee varus angles within the first 50% of the stance phase. No group differences were detected with regard to peak knee varus angles within the first 50% of the stance phase (Fig 3). Moreover, both KA groups negotiated decline walking with significant 27% (TKA, *p* = 0.001) and 22% (UKA, *p* = 0.007) reductions in first-peak knee adduction moments compared with the CG (Fig 4), accompanied by 14% lower adduction moment impulse values which did not reach statistical significance after post hoc analysis (*p* = 0.093^CG-TKA^, *p* = 0.072^CG-UKA^). The non-OP knee adduction moments and impulses of both KA groups were barely distinguished from the CG (Fig 4). No between-group or interlimb differences were observed concerning the FAP-CoM_add_ parameter, indicating that the subjects did not employ compensatory mechanisms, such as the Trendelenburg gait pattern.

## Discussion

It is stated that UKA provides several advantages over TKA in terms of, for example, better function, faster rehabilitation and participation in more sporting activities, but first and foremost, UKA might provide kinematics similar to those of the native knee [34–36]. However, there are only a few studies investigating patients after UKA, TKA, and healthy CGs by means of optoelectronic systems based on stereophotogrammetry. The main focus of the research has been on level walking and sagittal plane mechanics, whereas frontal and particularly transverse plane evaluations are often neglected. Therefore, the current study aimed to address the mentioned aspects in order to expose potential differences that might be related to different implant designs and locomotion tasks. To the author’s knowledge, this is the first study to include ramp negotiation tasks with patients after KA surgery.

### Transverse plane

The authors’ first hypothesis was not supported by the results: the selected parameters did not show significant differences in the transverse plane between the UKA and TKA groups at any time, irrespective of the locomotion task. However, the second hypothesis was supported by the results. Both KA groups presented similar discrepancies in comparison with the CG regarding certain parameters, particularly during decline walking. Nevertheless, the TKA group showed more considerable abnormalities in the OP knee presenting reduced peak knee internal rotation angles during all tasks. Especially, decline walking exposed the most striking deficits in the TKA group compared with the CG. Interestingly, all groups showed nearly equal internal rotation moments, accompanied by greater, but not significantly different, transverse knee joint stiffness values in both KA groups. Particularly noteworthy are the internal knee rotation curve shapes for the non-OP knees of both KA groups, which are similar to the curve shape of the CG (Fig 1). These results illustrate, besides reduced peak values and diminished RoM, significantly greater joint stiffness values in the TKA group’s OP knee than in the non-OP knee. Indeed, the Wilcoxon signed-rank test exposed no statistical difference (*p* = 0.06) due to the high standard deviation between the OP knee and non-OP knee of the UKA group according to joint stiffness. Nevertheless, the values of 0.065 Nm/° (OP knee) versus 0.011 Nm/° (non-OP knee) and the corresponding effect size (0.54) suggest a meaningful result. The results concerning the constrained internal knee rotation are in agreement with the previous work of McClelland et al. [37] who reported decreased knee internal rotation angles in TKA patients compared with a healthy CG during level walking. In this regard, Wünschel et al. [38] also observed in a cadaveric study lower internal rotation values in TKA knees during kneeling activities. The authors conjectured that TKA may impair tibial internal rotation due to the concave shape of the inlay.

On the other hand, this aspect could not explain the reduced internal rotation in the UKA group, neither in the current study during decline walking nor in the study by Argenson et al. [39], because the UKA-tibial inlays were flat in both studies. The authors assumed that progressive laxity of the ACL may occur over time in UKA patients [39]. Hence, neuromuscular factors could have a significant influence on the mentioned deficit, aside from different implant designs. It has been shown, that the hamstrings are able to rotate the tibia externally as well as inhibit anterior tibial translation in ACL-deficient knees by means of co-contraction [40,41]. Thus, due to the sloped ramp surface in the current study, increased tibial acceleration occurred in an anterior direction during decline walking compared with level walking (see supporting information, S2 Table). Consequently, greater co-contraction of the hamstrings was apparent during decline walking, particularly in the OP-knees of the TKA group, where the ACL was absent. Finally, the higher transverse joint stiffness values in the OP knee in contrast to the non-OP knee provide support for the above mentioned mechanism.

Although not calculated, the authors presume that increased coefficients of friction, which usually occur between a cobalt-chromium alloy and an ultra-high-molecular-weight polyethylene (UHMWPE) [42,43], might contribute to the impaired internal knee rotation of both KA groups in comparison with the non-OP knee and CG. Moreover, due to increased friction higher stress might occur on the interface between the tibial tray and bony structures. The extent of rotational load transmitted to the bone-implant interface is directly proportional to the frictional force. Due to rotational forces acting on the tibial tray, studies demonstrated torsional micromotion [28,44]. Transverse plane moments probably act like an unscrewing mechanism accompanied by axial and shear loads contributing to aseptic component loosening. In this regard, mobile-bearing TKA and UKA aim to improve survivorship rates providing more natural tibial internal rotation and reducing contact stresses compared with fixed-bearing implants by means of rotating platforms [45,46]. Nevertheless, mobile-bearing KA still remains controversial in terms of enhanced survivorship and more natural kinematics, essentially complicating the question to what extent the rotational behavior of the knee influences the lifespan of KA [47,48].

### Frontal plane

Similar to the results obtained in the transverse plane, the frontal plane results did not support the first hypothesis. No statistically significant differences between the TKA and UKA groups were observed concerning the selected parameters. In accordance with the second hypothesis, clear discrepancies were apparent, in particular during decline walking in both KA groups compared with the CG, where decreased first-peak knee adduction moments were present in the KA groups. The effect size of 0.36 clarifies a moderate clinical relevance in terms of reduced load in the medial compartment of the replaced knee, as the knee adduction moment is stated to be a surrogate indicator of load redistribution to the medial knee compartment [49,50]. Additionally, polyethylene wear primarily occurs in the medial compartment of the implants [51,52], although it should be considered that UHMWPE wear rates have been reduced in recent years due to improved mechanical properties of UHMWPE [26,27,53]. In the current study, knee adduction moments of both KA groups were significantly lower during decline walking but not different during the other investigated tasks compared with the CG. This suggests that a premature endoprosthesis failure due to wear of the medial implant compartment is unlikely. Studies have reported heterogeneous results concerning first-peak knee adduction kinetics in TKA patients, at least during level walking. In Benedetti et al. [54] TKA patients demonstrated significantly reduced knee adduction moments both 12 and 24 months post-surgery compared with a CG. The authors explained that measured compensatory mechanisms in the trunk could be responsible for the reduced knee adduction moments in the TKA group. Likewise, Urwin et al. [55] showed reduced knee adduction moments of TKA patients nine months post-surgery compared with controls. It is important to mention, that in both studies, the TKA patients performed level walking significantly more slowly than the appropriate CG, which could partly explain the mentioned differences. Contrarily, in the current study, no between-group differences were found with respect to gait velocity, regardless of the locomotion task. On the other hand, in a study by Worsley et al. [56], a mixed group consisting of TKA and UKA patients conducted level walking with slightly higher knee adduction moments compared with healthy controls (*p* = 0.27), whereby the non-OP knee showed higher values than the OP knee. In the current study, only the UKA group showed higher, but statistically not significantly different (*p* = 0.109), adduction moments in the OP knee during level walking. Interestingly, both KA groups accomplished decline walking with notably reduced knee adduction moments in their OP knee compared with the CG (η^2^ = 0.36). The non-OP knee exhibited greater knee adduction moments than the OP knee in either KA group (Fig 4) accompanied by greater varus angles in the non-OP knee (Fig 3). In this regard, it is important to mention that static reference trials exhibited normally aligned limbs and no interlimb asymmetries. However, knee adduction moments as well as adduction moment impulses of the non-OP knee did not exceed the CG’s values. Hence, reduced knee adduction moments in the affected knee do not necessarily result in a medial compartment overload of the contralateral non-replaced knee. In addition, compensatory mechanisms, such as increased lateral trunk sway in the frontal plane, which could explain the reduced knee adduction moments during decline walking in the OP knee, were not detected by means of the FAP-COP_add_ distance calculation.

Only a few studies included a CG or reported P-values. To the authors’ knowledge, there is no study investigating ramp negotiation with patients after TKA and UKA. Hence, it is difficult to integrate the results of the current study regarding sloped walking with the existing literature and therefore, comparisons should be treated with caution. However, the results of the current study clarify the importance of analyzing physically more demanding ADL, such as ramp negotiation, in addition to level walking. Although decline walking shows similar lower limb kinematics and kinetics to level walking [10,11], the knee joint seems to be mostly affected by negative inclinations, which is in accordance with previous literature. Thus, impairments after KA surgery should be more apparent during decline walking than level walking.

The present study has some limitations, especially with regard to the TKA group consisting of posterior stabilized and posterior cruciate ligament retained knee endoprosthesis. However, generally, no statistical differences in terms of functional and clinical parameters were detected in the previous literature. Nevertheless, the superiority of one of the prosthesis types remains controversial [57–59]. Data extracted from the transverse and frontal plane should be interpreted with caution due to its known sources of measurement and modeling issues [29,60]. In order to reduce bias due to high BMI values and associated increased soft-tissue artifacts, the authors excluded subjects with BMI values higher than 31. Furthermore, the authors placed particular importance on keeping the age of the subjects consistent (Table 1). The groups were not gender-matched, which may have affected the results of this study. Thus, on account of the strict inclusion criteria, it was not possible to include more subjects in the appropriate groups.

## Conclusion

The results of the current study are in accordance with the second hypothesis explaining the significant importance of including walking on sloped surfaces in biomechanical studies and rehabilitative treatment evaluation of KA patients. Particularly, decline walking exposed appreciable discrepancies in both KA groups compared with the CG presenting compromised knee internal rotation, especially in the TKA group. However, the non-OP knee showed similar kinematics in the transverse plane compared with the CG’s. Contrary to the authors’ first hypothesis, no statistically significant differences between the TKA and UKA groups in terms of the parameters evaluated and locomotion tasks were found. Both KA groups exhibited similar discrepancies in the OP knee compared with the CG as well as to the KA groups’ non-OP knee, in particular during decline walking. However, the TKA group presented abnormal knee biomechanics to a greater extent than the UKA group. The relatively small sample size or insufficient measuring sensitivity could be potential reasons for the absence of statistically significant differences between the TKA and UKA group.

## Supporting Information

S1 Table Sagittal plane knee kinematics and kinetics during level walking. *Indicates significant difference between corresponding groups. Peak values are presented for the first 50% of stance phase. Mean (Ø) flexion velocity is calculated from heel strike until maximum knee flexion for the first 50% of stance phase.
(PDF)

S2 Table Sagittal plane knee kinematics and kinetics during decline and incline walking. *Indicates significant difference between corresponding groups. Peak values are presented for the first 50% of stance phase. Mean (Ø) flexion velocity is calculated from heel strike until maximum knee flexion for the first 50% of stance phase.
(PDF)
