# Supplementary material for: Non-Sagittal Knee Joint Kinematics and Kinetics during Gait on Level and Sloped Grounds with Unicompartmental and Total Knee Arthroplasty Patients
Source: PLoS One. 2016 Dec 21;11(12):e0168566. doi: 10.1371/journal.pone.0168566 (PMC5176302; doi:10.1371/journal.pone.0168566)
Supplement: S1 Table — *Indicates significant difference between corresponding groups. Peak values are presented for the first 50% of stance phase. Mean (Ø) flexion velocity is calculated from heel strike until maximum knee flexion for the first 50% of stance phase. (PDF) [file pone.0168566.s001.pdf]

S1 Table. Sagittal plane knee kinematics and kinetics during level walking.

Level walking						
**Parameter**	**CG**	**TKA**	**UKA**	**p-value**	**Effect size**	**Power**
Flexion angle [°]	19.1 ± 4.7	15.1 ± 5.5	14.0 ± 3.4	0.93^{CG-TKA}<br>$0.02^{*CG-UKA}$<br>$0.84^{TKA-UKA}$	0.21	0.18
Ø Flexion velocity [°/s]	126.7 ± 27.0	88.7 ± 24.5	95.7 ± 21.4	$0.002^{*CG-TKA}$<br>$0.009^{*CG-UKA}$<br>$0.774^{*TKA-UKA}$	0.34	0.41
RoM [°]	17.4 ± 3.5	13.4 ± 3.0	14.5 ± 2.1	$0.05^{*CG-TKA}$<br>$0.046^{*CG-UKA}$<br>$0.636^{TKA-UKA}$	0.27	0.27
Flexion moment [Nm/(kg·m)]	0.42 ± 0.1	0.31 ± 0.1	0.39 ± 0.1	$0.03^{*CG-TKA}$<br>0.8^{CG-UKA}<br>$0.11^{TKA-UKA}$	0.2	0.16
Flexion mom. impulse [Nms/(kg·m)]	0.05 ± 0.02	0.04 ± 0.01	0.05 ± 0.02	0.160	0.09	0.07

*Indicates significant difference between corresponding groups. Peak values are presented for the first 50% of stance phase. Mean (Ø) flexion velocity is calculated from heel strike until maximum knee flexion for the first 50% of stance phase.
