# Supplementary material for: Non-Sagittal Knee Joint Kinematics and Kinetics during Gait on Level and Sloped Grounds with Unicompartmental and Total Knee Arthroplasty Patients
Source: PLoS One. 2016 Dec 21;11(12):e0168566. doi: 10.1371/journal.pone.0168566 (PMC5176302; doi:10.1371/journal.pone.0168566)
Supplement: S2 Table — *Indicates significant difference between corresponding groups. Peak values are presented for the first 50% of stance phase. Mean (Ø) flexion velocity is calculated from heel strike until maximum knee flexion for the first 50% of stance phase. (PDF) [file pone.0168566.s002.pdf]

S2 Table. Sagittal plane knee kinematics and kinetics during decline and incline walking.

	Decline walking						Incline walking					
Parameter	CG	TKA	UKA	p-value	Effect size	Power	CG	TKA	UKA	p-value	Effect size	Power
Flexion angle [°]	31.8 ± 4.5	27.1 ± 4.6	25.9 ± 4.1	0.039*CG-TKA<br>0.006*CG-UKA<br>0.785TKA-UKA	0.27	0.27	36.8 ± 6.0	34.0 ± 5.3	33.8 ± 5.8	0.371	0.06	0.06
Ø Flexion velocity [°/s]	153.8 ± 35.4	118.0 ± 30.4	116.7 ± 27 8	0.029*CG-TKA<br>0.016*CG-UKA<br>0.984TKA-UKA	0.25	0.24	81.4 ± 51.8	62.3 ± 44.15	72.7 ± 36.3	0.398	0.03	0.05
RoM [°]	26.1 3.6 ±	27.3 ± 8.1	29.5 ± 6.7	0.405	0.05	0.06	22.2 ± 4.4	23.3 ± 6.4	21.7 ± 4.5	0.692	0.02	0.05
Flexion moment [Nm/(kg·m)]	0.69 ± 0.2	0.48 ± 0.1	0.55 ± 0.1	0.005*CG-TKA<br>0.063*CG-UKA<br>0.534TKA-UKA	0.27	0.27	0.52 ± 0.1	0.39 ± 0.1	0.48 ± 0.1	0.037*CG-TKA<br>0.733CG-UKA<br>0.154TKA-UKA	0.18	0.14
Flexion mom. impulse [Nms/(kg·m)]	0.11 ± 0.04	0.08 ± 0.02	0.09 ± 0.02	0.209	0.5	0.74	0.08 ± 0.03	0.07 ± 0.03	0.09 ± 0.02	0.233	0.1	0.08

*Indicates significant difference between corresponding groups.

Peak values are presented for the first 50% of stance phase. Mean (Ø) flexion velocity is calculated from heel strike until maximum knee flexion for the first 50% of stance phase.
